# Supplementary material for: Demographically explicit scans for barriers to gene flow using gIMble
Source: PLoS Genet. 2023 Oct 10;19(10):e1010999. doi: 10.1371/journal.pgen.1010999 (PMC10610087; doi:10.1371/journal.pgen.1010999)
Supplement: S1 Text — The supporting information is organized into three sections: (1) Supplementary methods; gIMble commands used for the Heliconius analysis. (2) Supplementary tables A—C (3) Supplementary Figures A—M. (PDF) [file pgen.1010999.s001.pdf]

Supplementary Text 1: Demographically explicit scans for barriers to gene flow using gIMble

Dominik R Laetsch[1,☯], Gertjan Bisschop[1,☯], Simon H Martin[1], Simon Aeschbacher[2], Derek Setter[1] and Konrad Lohse[1,*]

1 Institute of Ecology and Evolution, University of Edinburgh, Edinburgh, UK
2 Department of Evolutionary Biology and Environmental Studies, University of Zurich, Zurich, Switzerland

☯These authors contributed equally to this work.
* konrad.lohse@ed.ac.uk

Contents

1 Supplementary methods **3**

2 Supplementary tables **6**

3 Supplementary figures **9**

List of Tables

A *Heliconius* Illumina read sets . . . 6
B Estimates of the demographic history of *H. melpomene* and *H. cydno* . . . 7
C Effect of block length on demographic parameter estimates . . . 8

List of Figures

A `gIMble` workflow . . . 9
B Effect of block length on bSFS configuration probabilities . . . 10
C Window span distribution . . . 11
D Pairwise divergence vs. mean heterozygosity . . . 12
E d_{xy} and F_{ST} along the genome . . . 13
F Best supported global demographic history . . . 14
G N_e vs. chromosome length . . . 15
H F_{ST} vs. recombination rate . . . 16
I N_e and m_e vs. recombination rate and CDS density . . . 17
J Goodness-of-fit of the aggregate migration–selection model of Aeschbacher et al. 2017 18
K Local point estimates of m_e and $\Delta_{B,0.2}$ . . . 19
L Impact of window size on the power to detect a local reduction in m_e . . . 20
M Impact of r_{bp} on the power to detect a local reduction in m_e . . . 21

1 Supplementary methods

```
# gimble parse (elapsed: 00h:22m:30.888s)
~/gIMble/gimble parse \
    -g input/heliconius_20220202.genomefile \
    -v input/heliconius_20220202.vcf.gz \
    -b input/heliconius_20220202.non_genic_non_repeats.bed \
    -s input/heliconius_20220202.samples.csv \
    -z heliconius.v1

# gimble blocks (elapsed: 01h:25m:29.398s)
~/gIMble/gimble blocks -z heliconius.v1.z -l 64

# gimble windows (elapsed: 00h:34m:07.976s)
~/gIMble/gimble windows -z heliconius.v1.z -w 50000 -s 10000

# gimble tally
## tally of blocks (elapsed: 00h:07m:43.094s)
~/gIMble/gimble tally -z heliconius.v1.z -d blocks -l blocks
## tally of windows (elapsed: 00h:32m:06.685s)
~/gIMble/gimble tally -z heliconius.v1.z -d windows -l windows

# gimble optimize

## optimize windows-windowsum tally under model DIV (elapsed: 00h:03m:48.604s)
~/gIMble/gimble optimize -z heliconius.v1.z -d tally/windows -w \
    -r A -u 2.9e-9 -g CRS2 -p 1 -s midpoint -i 10000 \
    -A=100_000,3_000_000 -B=100_000,3_000_000 \
    -C=100_000,3_000_000 -T=0,5_000_000 -m DIV -l DIV

## optimize windows-windowsum tally under model MIG_AB (elapsed: 00h:03m:15.508s)
~/gIMble/gimble optimize -z heliconius.v1.z -d tally/windows -w \
    -r A -u 2.9e-9 -g CRS2 -p 1 -s midpoint -i 10000 \
    -A=100_000,3_000_000 -B=100_000,3_000_000 \
    -M=1e-10,2.21E-06 -m MIG_AB -l MIG_AB

## optimize windows-windowsum tally under model MIG_BA (elapsed: 00h:03m:14.747s)
~/gIMble/gimble optimize -z heliconius.v1.z -d tally/windows -w \
    -r A -u 2.9e-9 -g CRS2 -p 1 -s midpoint -i 10000 \
    -A=100_000,3_000_000 -B=100_000,3_000_000 \
    -M=1e-10,2.21E-06 -m MIG_BA -l MIG_BA

## optimize windows-windowsum tally under model IM_AB (elapsed: 01h:38m:10.970s)
~/gIMble/gimble optimize -z heliconius.v1.z -d tally/windows -w \
    -r A -u 2.9e-9 -g CRS2 -p 1 -s midpoint -i 10000 \
```

```
    -A=100_000,3_000_000 -B=100_000,3_000_000 \
    -C=100_000,3_000_000 -T=0,5_000_000 \
    -M=0,2.21E-06 -m IM_AB -l IM_AB

## optimize windows-windowsum tally under model IM_BA (elapsed: 00h:47m:37.386s)
~/gIMble/gimble optimize -z heliconius.v1.z -d tally/windows -w \
    -r A -u 2.9e-9 -g CRS2 -p 1 -s midpoint -i 10000 \
    -A=100_000,3_000_000 -B=100_000,3_000_000 \
    -C=100_000,3_000_000 -T=0,5_000_000 \
    -M=0,2.21E-06 -m IM_BA -l IM_BA

# gimble makegrid (elapsed: 00h:06m:47.222s)
~/gIMble/gimble makegrid -z heliconius.v1.z -m IM_BA \
    -b 64 -r A -u 2.9e-9 -k 2,2,2,2 \
    -A=200_000,3_000_000,12,lin \
    -B=100_000,2_000_000,12,lin \
    -C 100_000,2_000_000,12,lin \
    -T 4_256_034 -M 0,2.21E-06,16,lin \
    -p 48 -e 19 -l IM_BA_final

# gimble gridsearch of windows (elapsed: 00h:01m:35.828s)
~/gIMble/gimble gridsearch -z heliconius.v1.z \
    -g makegrid/IM_BA_final -d tally/windows \
    -p 50 -c 1000

# gimble simulate

## gimble simulate of best model

## gimble simulate of unconstrained gridsearch result (elapsed: 16h:24m:34.610s)
~/gIMble/gimble simulate -z heliconius.v1.z \
    --seed 19 --replicates 100 --windows 11217 --blocks 500 \
    --block_length 64 -a 10 -b 10 \
    --gridsearch_key gridsearch/windows/IM_BA_final \
    --rec_map heliconius.v1.rec_final_Hmel_1000kb_2023.txt \
    -k 2,2,2,2 -s IM_BA_grid_unconstrained -p 55 \
    -u 2.9e-9

## gridsearch of simulation (elapsed: 03h:00m:43.823s)
~/gIMble/gimble gridsearch -z heliconius.v1.z \
    -g makegrid/IM_BA_final \
    -d simulate/IM_BA_grid_unconstrained \
    -p 10 -c 500

## gimble simulate of  constrained gridsearch result (elapsed: 21h:27m:05.082s)
```

```
~/gIMble/gimble simulate -z heliconius.v1.z \
    --seed 19 --replicates 100 --windows 11217 --blocks 500 \
    --block_length 64 -a 10 -b 10 \
    --gridsearch_key gridsearch/windows/IM_BA_final \
    -t me=7.366666666666667e-07 \
    --rec_map heliconius.v1.rec_final_Hmel_1000kb_2023.txt \
    -k 2,2,2,2 -s IM_BA_grid_constrained \
    -p 30 -u 2.9e-9

## gridsearch of simulation (elapsed: 07h:41m:01.082s)
~/gIMble/gimble gridsearch -z heliconius.v1.z \
    -g makegrid/IM_BA_final \
    -d simulate/IM_BA_grid_constrained \
    -p 10 -c 500
```

Listing 1. gIMble commands

2 Supplementary tables

Table A. *Heliconius* Illumina read sets downloaded from ENA. COV-mean and COV-sd represent mean and standard deviation of read coverage when mapping quality-trimmed reads against the Hmel2.5 assembly.

Read group ID	ENA run accession	sex	taxon	COV-mean	COV-sd
chi.CAM25091.f	ERR2298220	F	*H. cydno chioneus*	27.71	78.67
chi.CAM25137.f	ERR2298222	F	*H. cydno chioneus*	29.6	77.41
chi.CAM580.m	ERR2298196	M	*H. cydno chioneus*	23.41	44.5
chi.CAM582.m	ERR2298197	M	*H. cydno chioneus*	25.7	48.83
chi.CAM585.m	ERR2298199	M	*H. cydno chioneus*	24.96	52.37
chi.CAM586.m	ERR2298200	M	*H. cydno chioneus*	24.3	49.73
chi.CJ553.m	ERR260295	M	*H. cydno chioneus*	37.62	68.71
chi.CJ560.m	ERR260296	M	*H. cydno chioneus*	37.11	67.13
chi.CJ564.m	ERR260297	M	*H. cydno chioneus*	41.27	78.71
chi.CJ565.m	ERR260298	M	*H. cydno chioneus*	48.36	76.16
ros.CAM1841.m	ERR2298205	M	*H. melpomene rosina*	28.7	47.35
ros.CAM1880.m	ERR2298206	M	*H. melpomene rosina*	31.94	52.82
ros.CAM2045.m	ERR2298208	M	*H. melpomene rosina*	23.19	30.51
ros.CAM2059.m	ERR2298209	M	*H. melpomene rosina*	26.35	34.71
ros.CAM2519.m	ERR2298210	M	*H. melpomene rosina*	28.48	31.07
ros.CAM2552.m	ERR2298211	M	*H. melpomene rosina*	25.16	56.86
ros.CJ2071.m	ERR260280	M	*H. melpomene rosina*	38.2	57.43
ros.CJ531.m	ERR260277	M	*H. melpomene rosina*	28.26	44.68
ros.CJ533.m	ERR260278	M	*H. melpomene rosina*	28.27	43.12
ros.CJ546.m	ERR260279	M	*H. melpomene rosina*	27.97	45.36

Table B. Estimates of the demographic history of *H. melpomene* and *H. cydno* inferred using `gIMble` *optimise* under models allowing either migration only (MIG), strict isolation (DIV) or isolation with migration (IM). The results based on the sum of window-wise bSFS tallies (windowsum) are near identical to the bSFS tally of all blocks (blocks). By default `gIMble` parameter estimates are scaled in absolute units, i.e. number of individuals and generations given a user specified μ. We have converted T to millions of years (MYA) assuming four generations per year.

model	$\ln CL$	N_{mel}	N_{cyd}	N_{anc}	T (MY)	m
blocks						
DIV	$-394,846,023$	1.12×10^6	1.49×10^6	1.42×10^6	0.243	n/a
$MIG_{\rightarrow cyd}$	$-395,756,127$	1.255×10^6	6.96×10^5	n/a	n/a	7.86×10^{-7}
$MIG_{\rightarrow mel}$	$-393,957,100$	0.529×10^5	1.29×10^6	n/a	n/a	7.80×10^{-7}
$IM_{\rightarrow cyd}$	$-394,051,624$	1.14×10^6	1.40×10^6	1.40×10^6	0.261	7.50×10^{-8}
$IM_{\rightarrow mel}$	$-393,767,380$	5.45×10^5	1.41×10^6	9.23×10^5	1.064	7.38×10^{-7}
windowsum						
DIV	$-391,893,600$	1.13×10^6	1.50×10^6	1.42×10^6	0.243	n/a
$MIG_{\rightarrow cyd}$	$-392,780,725$	1.26×10^6	7.00×10^5	n/a	n/a	7.91×10^{-7}
$MIG_{\rightarrow mel}$	$-391,030,511$	5.33×10^5	1.30×10^6	n/a	n/a	7.85×10^{-7}
$IM_{\rightarrow cyd}$	$-391,889,506$	1.16×10^6	1.41×10^6	1.40×10^6	0.263	8.30×10^{-8}
$IM_{\rightarrow mel}$	$-390,836,229$	5.49×10^5	1.415×10^6	9.28×10^5	1.054	7.41×10^{-7}

Table C. Estimates of the demographic history of *H. melpomene* and *H. cydno* inferred using `gIMble` *optimise* for varying block lengths (l) under the best fitting isolation with migration ($IM_{\rightarrow mel}$) model. By default, `gIMble` parameter estimates are scaled in absolute units, i.e. number of individuals and generations given a user specified μ. We have converted T to millions of years (MYA) assuming four generations per year.

l (bases)	N_{mel}	N_{cyd}	N_{anc}	T (MY)	m
48	6.26×10^5	1.47×10^6	6.92×10^5	0.888	6.25×10^{-7}
64	5.45×10^5	1.41×10^6	9.23×10^5	1.064	7.38×10^{-7}
128	5.15×10^5	1.37×10^6	10.4×10^5	1.221	7.82×10^{-7}

3 Supplementary figures

Fig A. `gIMble` workflow. Numbers correspond to the order of steps in the *Heliconius* analysis: `preprocess` (0) assures input data meet standard filters; `parse` (1) reads data into a `gIMble` store, the central data structure that holds all subsequent analysis. The modules `blocks` (2) and `windows` (3) partition the data. The data are then summarised as a `tally` (4) of blockwise mutation configurations (bSFSs) either across all pair-blocks (**blocks tally**) or for pair-blocks in windows (**windows tally**). Tallies may be used either in a bounded search of parameter space via the module `optimize` (5) or to evaluate likelihoods over a parameter grid (which is precomputed using `makegrid`, 6) via the `gridsearch` module (7). The `simulate` module (8) allows coalescent simulation of tallies (**simulate tally**) based on inferred parameters (using either global estimates or the gridsearch results for `window`-wise data). Simulated data can be analysed to quantify the uncertainty and/or potential bias of parameter estimates. The results held within a `gIMble` store can be described, written to column-based output files or removed using the modules `info` (9), `query` (10), and `delete` (11).

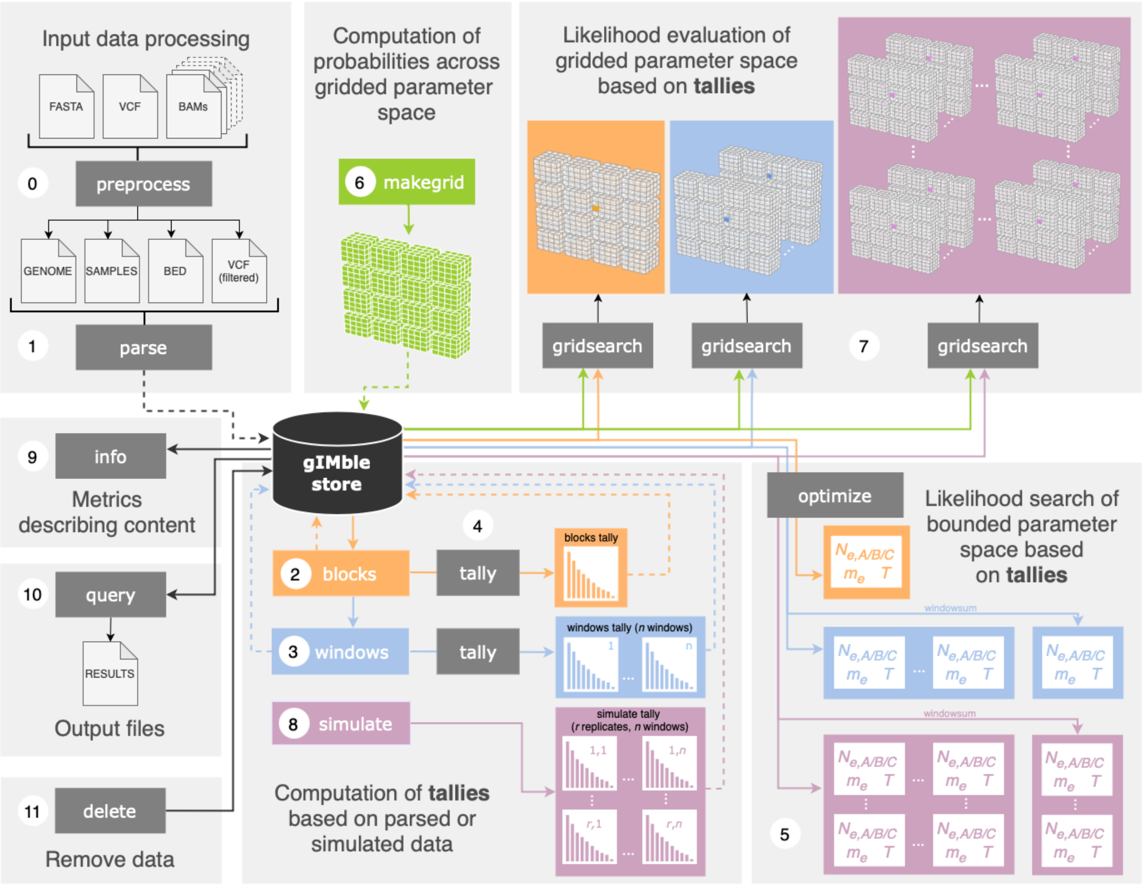

Fig B. The effect of block length on bSFS configuration probabilities. Intergenic sequence of the *Heliconius* genome was partitioned into pair-blocks of a given length l (x-axis). The y-axis shows for each choice of l the proportion of blocks that either carry a given number of mutations or fail the 4-gamete test. The total amount of blockable data is listed on top. Given our choice of $l = 64$ bases for the *Heliconius* analysis, about 50% of pair-blocks contain more than one variant. Partitioning the data into larger blocks reduces the size of the dataset and leads to biases as larger blocks are more likely to violate the 4-gamete test and be excluded from the inference.

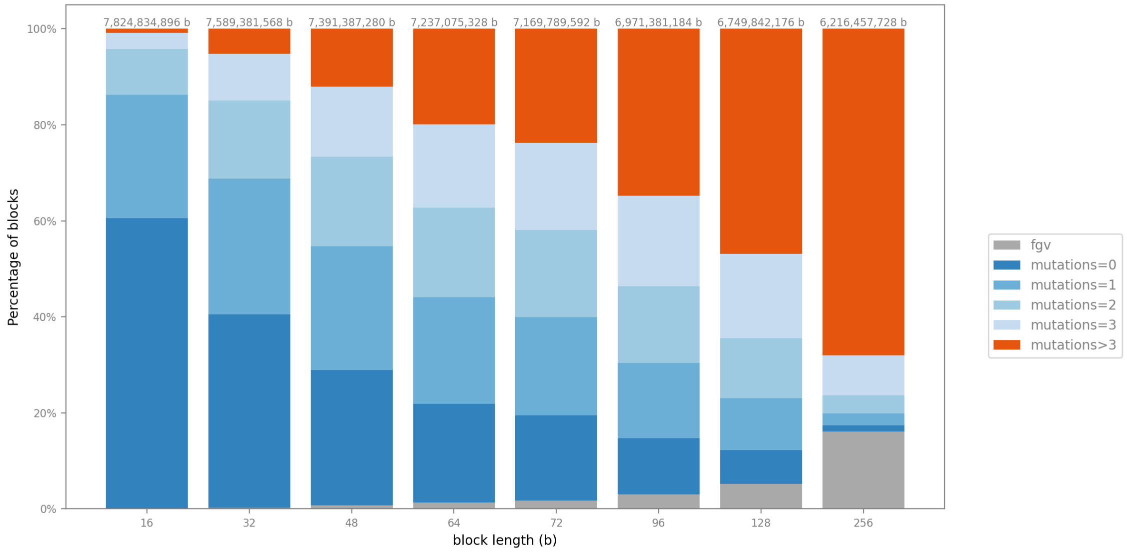

Fig C. The window span distribution. The *Heliconius* genome was analysed in sliding windows comprised of $L = 50,000$ (64 base) pair-blocks of intergenic sequence. Since each pair-block is sampled in a single heterospecific pair of individuals, this corresponds to a minimum window span of $(L * 64)/(n_A * n_B) = 32kb$ (black dashed line) given our sampling of $n_A = n_B = 10$ individuals each from *H. melpomeme* and *H. cydno*. Windows show a wide distribution of spans with a median (103 kb shown in red) substantially greater than this minimum.

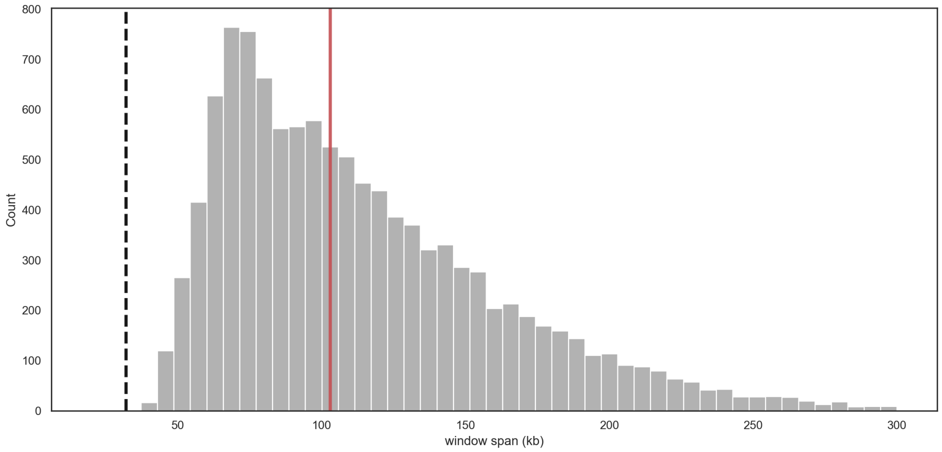

Fig D. The pairwise divergence (d_{xy}) for intergenic windows plotted against the corresponding mean heterozygosity H measured in *H. cydno* (left) and *H. melpomene* (right). Windows in the upper 5 % tail of window-wise F_{ST} are shown in blue.

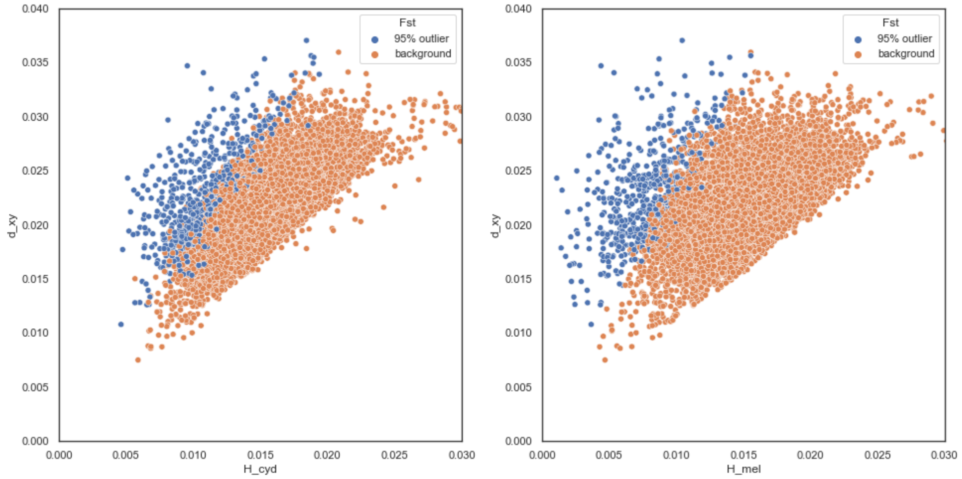

Fig E. Top: The divergence (d_{xy}, black), heterozygosity in *H. cydno* (green) and *H. melpomene* (blue) in windows of intergenic sequence (50,000 $\times$ 64 base pair-blocks) along the genome. Mean values are shown as horizontal lines. **Bottom**: The corresponding F_{ST} computed for each window using the summaries above. The upper 5 % tail of window-wise F_{ST} is delineated by the black dashed line, and the median is shown in gray.

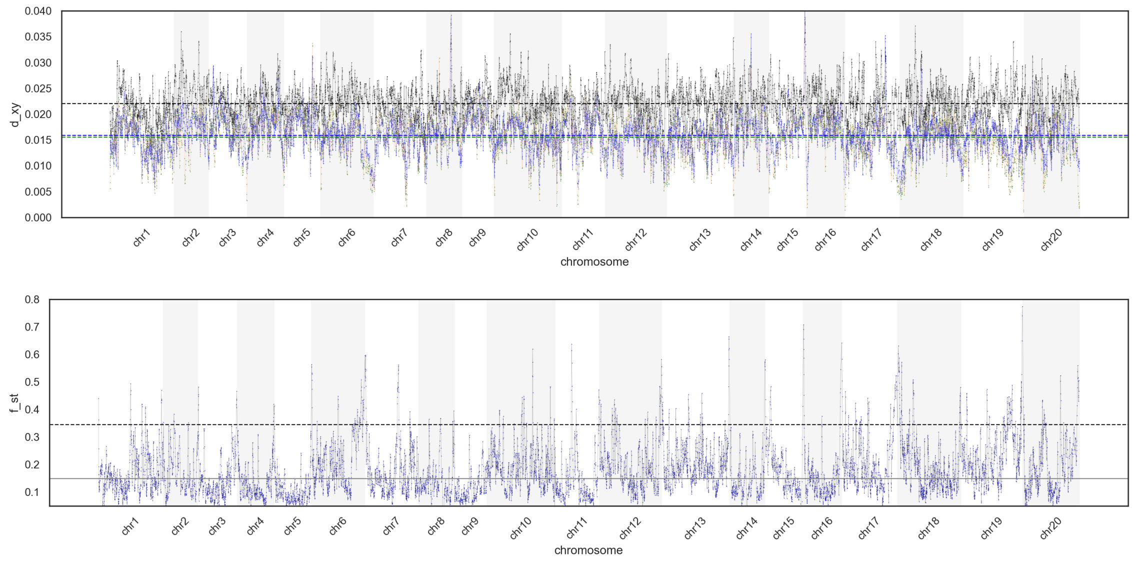

Fig F. The best supported global demographic history of Isolation and Migration (IM) between *H. melpomene* and *H. cydno* inferred using `gIMble optimize`.

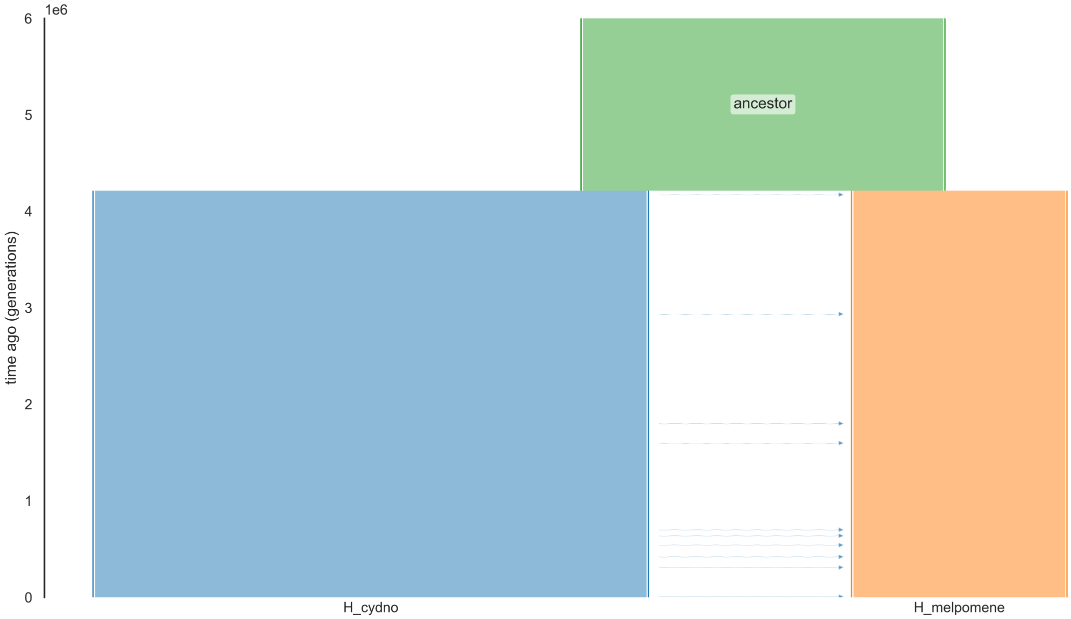

Fig G. Local estimates of N_e in *H. melpomene* (left) and *H. cydno* (right) are negatively correlated with chromosome length.

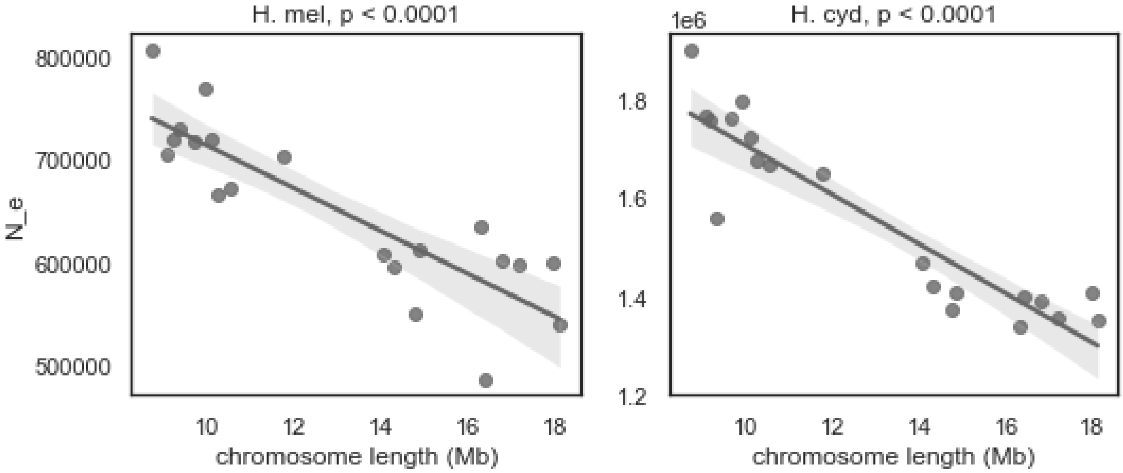

Fig H. The window-wise F_{ST} between *H. melpomene* and *H. cydno* is negatively correlated with the recombination rate.

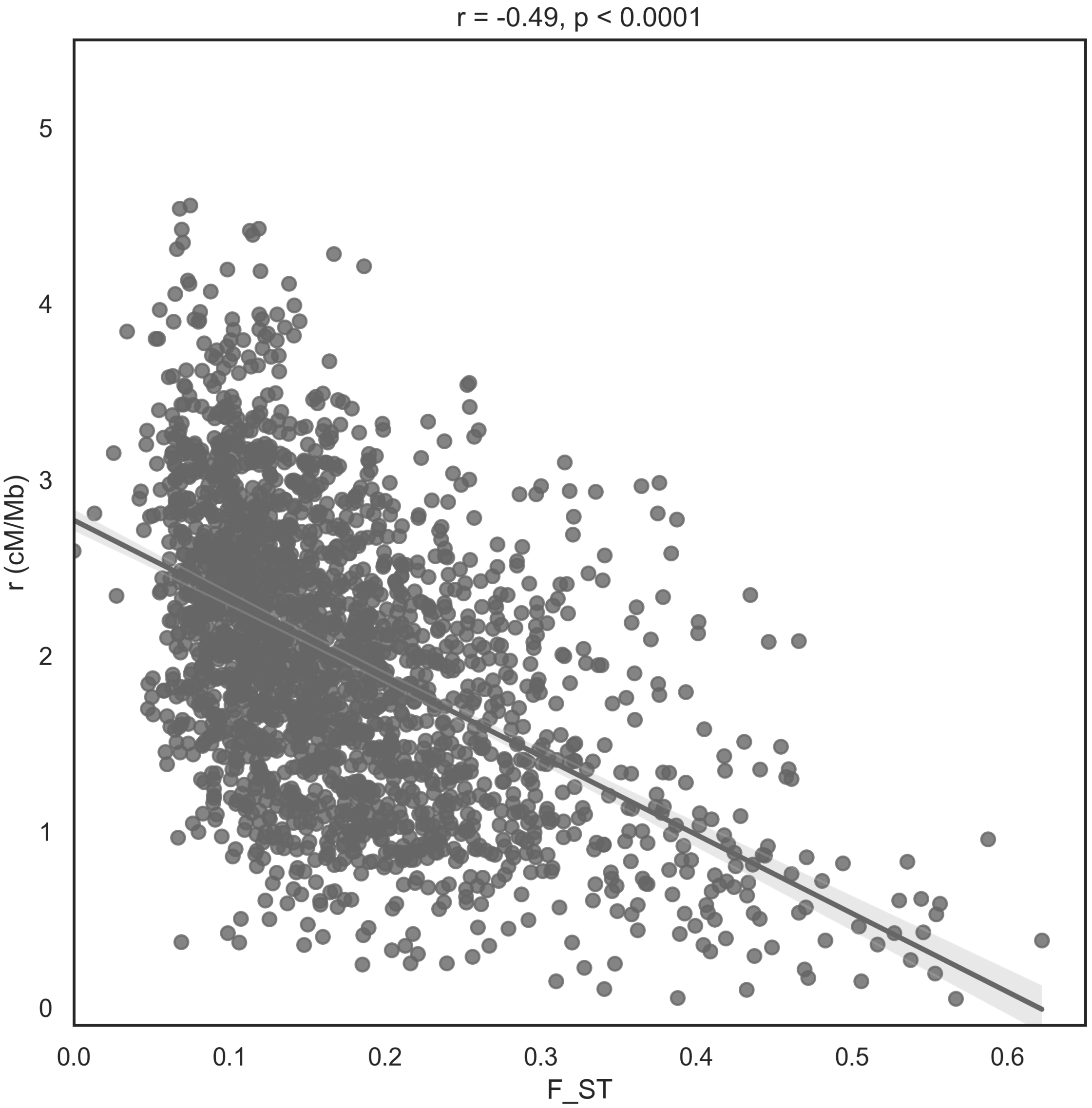

Fig I. Estimates of local N_e in *H. melpomene* and *H. cydno* and m_e are correlated positively with the recombination rate (top) and negatively with the density of CDS (bottom).

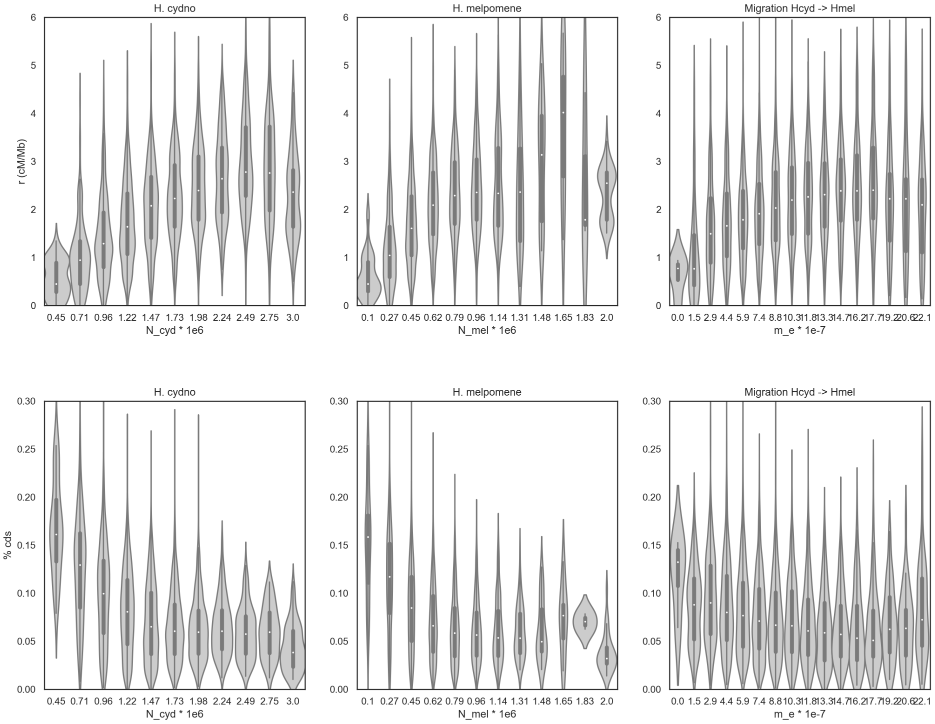

Fig J. The goodness-of-fit of the aggregate migration–selection model of Aeschbacher et al. [1] at various estimation ranges for the recombination rate (x-axis) and percentage of CDS (y-axis) when using the m_e estimates inferred for *H. melpomene* and *H. cydno* using `gIMble`. The goodness-of-fit is expressed both in terms of the residual standard error (left) and the natural logarithm of the difference in AIC from the best-fitting model (shifted by 1 to avoid indefinite values; right). Here, darker shading indicates a better fit.

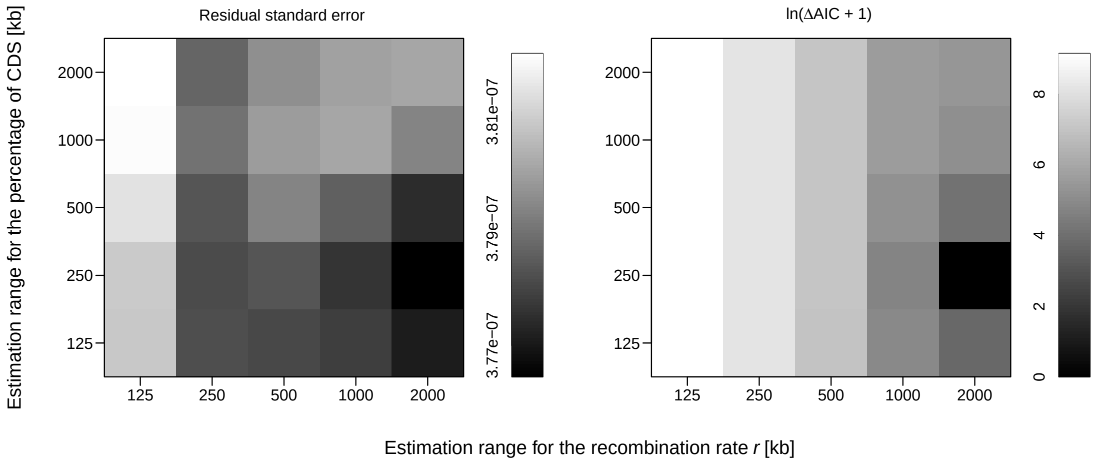

Fig K. Local point estimates of m_e (middle of each subplot) and $\Delta_{B,0.2}$ (bottom of each subplot) for the three chromosomes containing large effect wing patterning genes. Results from the (best-fitting) $IM_{\rightarrow mel}$ model are in black, results from the $MIG_{\rightarrow mel}$ model are in blue, and the predicted m_e under the polygenic model (modified from [1]) is shown in green. For each chromosome, the $\Delta_{B,0.2} > 0$ barriers under the $IM_{\rightarrow mel}$ model are shown in red (top of each subplot). $\Delta_{B,0.2}$ identifies all three known wing pattern genes as barriers (dark blue):*wnt-A*, *cortex* and *optix*) on chromosomes 10 (top), 15 (centre) and 18 (bottom), respectively. Given that causal sites may be situated in regulatory regions, which in the case of *optix* extends $\approx$ 100 kb away from the gene [2], we have highlighted the 100 kb up and downstream in lighter blue. In orange, we highlight an additional $\Delta_{B,0.2} > 0$ barrier on chromosome 18 that coincides with the *Grik2* and *regucalcin2* that are associated with male wing pattern preference.

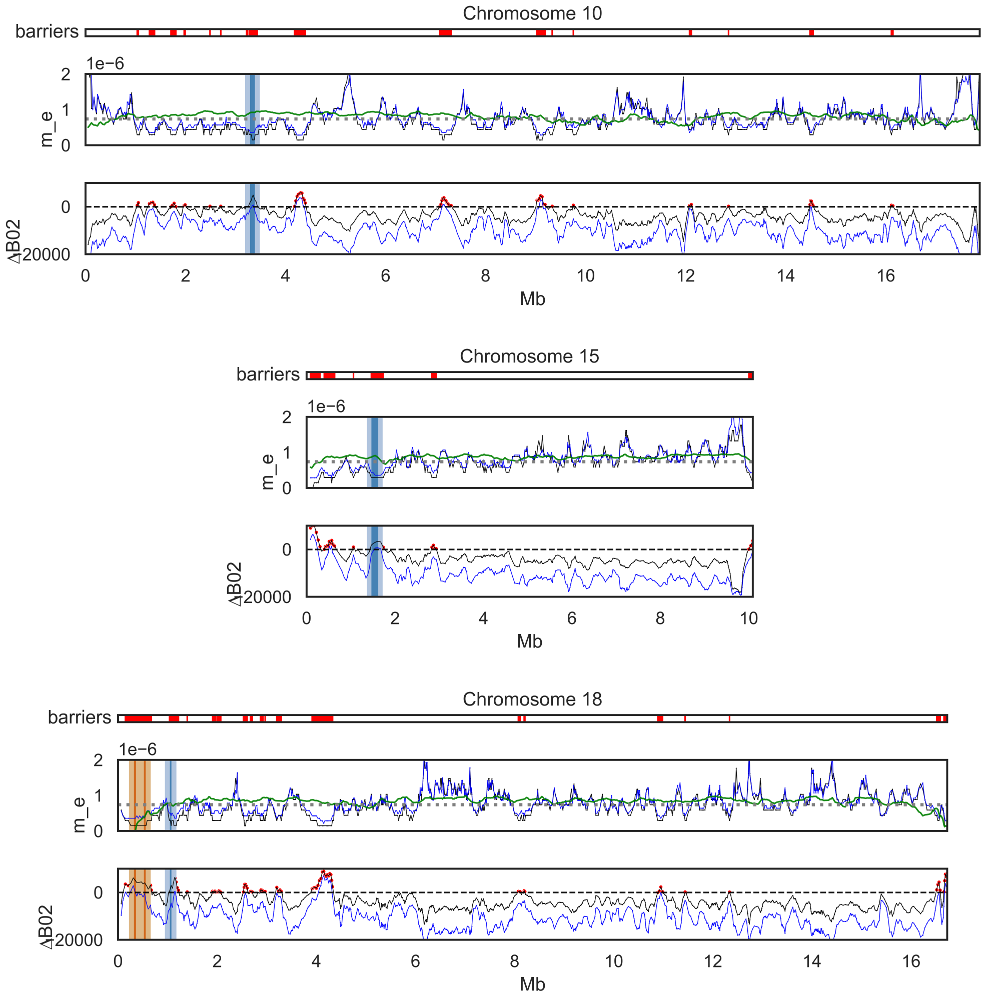

Fig L. The impact of window size on the power to detect a local reduction in m_e relative to the estimated background migration rate. The black line corresponds to the window size used in the *Heliconius* analysis.

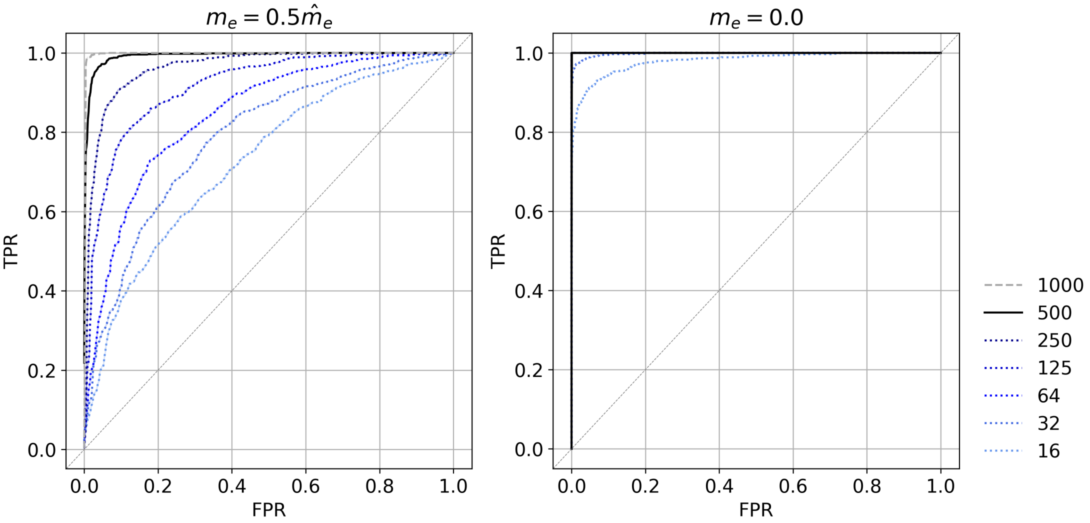

Fig M. The impact of r_{bp} on the power to detect a local reduction in m_e relative to the estimated background migration rate. The black line indicates the average recombination rate in *Heliconius*.

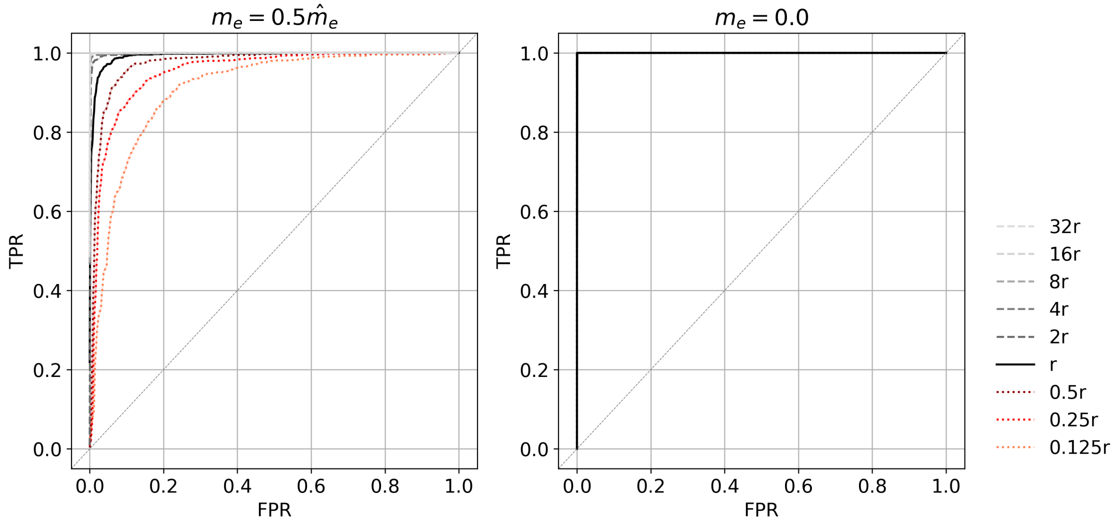

References

1. Aeschbacher S, Selby JP, Willis JH, Coop G. Population-genomic inference of the strength and timing of selection against gene flow. Proceedings of the National Academy of Sciences. 2017;114(27):7061–7066. Available from: http://www.pnas.org/lookup/doi/10.1073/pnas.1616755114.

2. Wallbank RWR, Baxter SW, Pardo-Diaz C, Hanly JJ, Martin SH, Mallet J, et al. Evolutionary Novelty in a Butterfly Wing Pattern through Enhancer Shuffling. PLoS Biology. 2016;14(1):1–16.
