# Supplementary material for: Demographically explicit scans for barriers to gene flow using gIMble
Source: PLoS Genet. 2023 Oct 10;19(10):e1010999. doi: 10.1371/journal.pgen.1010999 (PMC10610087; doi:10.1371/journal.pgen.1010999)
Supplement: Attachment — Submitted filename: gimble.pdf [file pgen.1010999.s002.pdf]

Review of: Demographically explicit scans for barriers to gene flow using gIMble

Speciation genomic studies primarily aim to understand the genomic architecture of barriers to gene flow, including the number, distribution and effect size of barrier loci. Achieving these aims requires that researchers are able to quantify how rates of gene flow vary across genomes, while also accounting for the diverse array of other factors that can shape patterns of variation. Methodologies for addressing these aims have lagged far behind access to data. In this manuscript, Laetsch et al. outline and implement a new model-based inference scheme that represents a significant leap beyond what is currently available.

This is really beautiful paper that not only introduces the method and how to use it, but applies it to a well know study system. I can really see that the authors have tried to make this paper accessible to broad readership, but in revising it, I would urge them to take a step back and really think carefully how they would explain things to a new researcher entering the field. You think of the following comments as a set of clarifications, which the authors could try and expand on.

Minor comments:

Line 94: maybe replace with 'so does not reveal the locations of barrier loci in the genome', which is a bit clearer than 'does not allow scanning for barriers to gene flow'.

Paragraph 69: I think it would be worth pointing out that efforts have been made to gauge heterogeneity in m_e (i.e., by modelling multiple classes of loci with different rates of m) while trying to control for confounding factors (i.e., by modelling classes of loci with different N_e), but this is crude and again does not reveal the locations of barrier loci.

Line 96: 'an awkward choice', makes it sound like researchers do one or the other, when many researchers just do both things (i.e., most researchers see them as complimentary rather than as alternatives), and then interpret the results in context of one another (i.e., comment on both the population processes and the heterogeneity). So this becomes a bit of a weak justification for why we would ideally like to reconstruct a fully specified model of the speciation process.

Line 109 on: this is the detail that I though was missing from the section 'model-based demographic inference' above.

Line 114: delete 'alternative'.

Line 156: invites the question ‘is variation in m_e correlated with recombination rate variation.

Line 169: conditioning the inference on global estimate of $\boldsymbol{T}$ is a reasonable simplification in a complex analysis like this. However, given that speciation often proceeds though selection on old variation, it makes me wonder when this would break down and what parameters might be affected. For example, old inversion polymorphisms or introgressed alleles may have much deeper divergence than the rest of genome in a local speciation event. I guess situations like this would be unlikely to effect the detection of barriers but would likely impact the estimates of m_e?

Fig 1: this is really helpful. But I wonder if you could add a simple cartoon of a demographic model here so less seasoned readers can see how the parameters map onto it.

Line 199: ‘we focused all analyses on intergenic sequence defined as any sequence without gene or repeat annotation’. Can you explain why you don’t you don’t include variable sites with gene annotation?

Line 200: ‘For the sake of simplicity, we ignore the Z chromosome, which is known to have greatly reduced genetic diversity and gene flow in Heliconius taxa’. Can you explain why this is simpler? Seems like an interesting region to include because there are some predictions how N_e would vary compared with other LGs.

Line 202: are the saying that the pair blocks all contain 64 variant sites when you say ‘bases’? Worth trying to keep this consistent. If so, what is the mean length, s.d., min, max of the pair blocks?

Line 219: So gene flow in the global model, migration only occurs in 1 direction (i.e., kind of a source sink model). Is this a intrinsic limitation of the method, or is it just something you decided to do here to keep the number of estimated parameters low? Also, I wonder if it’s useful to show fig S4 in the main text, perhaps coupled with table 2.

Line 262: what do negative values of ΔB,0 mean?

Fig 3 results. I’m surprised that so much of the genome shows really negative ΔB,0. Based on the explanation, I would have expected most windows to be hovering around ‘sea level’, and barrier regions to rise above it (which they do). Clarifying the above point will help.

Fig 5 caption: “the prediction under a polygenic model (modified from [9]) is shown in green”. This needs more context.

Fig. 7. Not a very intuitive layout (i.e., comparing x-axes between plots arrayed horizontally.). Each row would be better presented as a separate boxplot box/dot plots, so that the distributions could be more easily compared.

Line 356: this is a really fun section. Going into it, I really thought the answer would be counting up the number of barriers identified by Gimble. I think you need to start this section, explaining why it does not make sense to do this, because I think users of the program may be tempted to.

412-415: this is more clearly stated than in the introduction.

449: As I mentioned, I think this assumption should be stated way earlier.

491: Again this kind of explanation was needed near line 356.
