# Supplementary material for: Demographically explicit scans for barriers to gene flow using gIMble
Source: PLoS Genet. 2023 Oct 10;19(10):e1010999. doi: 10.1371/journal.pgen.1010999 (PMC10610087; doi:10.1371/journal.pgen.1010999)
Supplement: Attachment — Submitted filename: renamed_0aaa7.pdf [file pgen.1010999.s003.pdf]

gIMble reviewer’s responses

Reviewer #1

The authors presented here a new method to detect genetic barriers between recently diverged species. The method builds upon their previous work, using coalescent theory and the site-frequency spectrum as the main statistic. The method uses a two-step approach: first population sizes, divergence time and gene flow between the two species are estimated genome wide. In a second time, all parameters but divergence time are estimated locally, with regions with a local migration rate of 0 (or below a certain threshold) interpreted as harboring genetic barriers. The method is illustrated in the case of Heliconius, with the authors recovering regions already known for harboring genetic barriers to gene flow.

The authors presented here a new approach that is nicely illustrated using Heliconius. The manuscript is in general clear and well written (but see point below).

Comments:
The authors followed the guidelines of PlosGen to have the methods at the end of the manuscript, but in my opinion, this decreases the readability of the manuscript. I would consider having a small **model section** first to explain the model being used here (i.e. how blocks and windows are defined and what the bSFS is.). In general, I found the details on how the different elements were defined a bit sparse.

We thank the reviewer for this excellent suggestion, which is along the same lines as many of the comments raised by reviewer 3. We have restructured the MS and added subsections entitled “The model” and “Summarising genomic variation in blocks and windows” at the beginning of the Results as suggested.

- It was unclear to me how blocks were defined in the first place, in particular, whether the 64 bp have to be contiguous or not. I only found the answer that they do not have to be on line 698, because of the existence of the block_span option in addition to the block_length one. In addition, it is also not specified whether there is a minimum level of polymorphism expected for a given block. Within a block, can 30 bp be skipped because of coverage issue in one individual, or is it only indels and multiallelic sites that are skipped. Based on the definition of the bSFS (l 617 to 622), having a block without any polymorphism seems impossible, but it is unclear to me how such a case is handled.

We have tried to explain much earlier and more clearly how blocks are defined in the model section “Summarising genomic variation in blocks and windows” (l. 180).

- Windows are defined over 500 blocks but not over an uninterrupted stretch of DNA. Are there any conditions on the “missing” parts or can it be anything (including genes, small inversions, hot spot of recombination…). As a follow-up, can we therefore have genes within a window, or can they only be found outside windows.

Windows can span any unsampled data partition, e.g. in our analysis of intergenic data, windows extend across masked genic regions. We have made this clear in the new section (see our response to the previous comment).

- It would be nice to give a quick explanation (as in line 617 to 622) of what the bSFS is when it is introduced.

A great suggestion. We have included this in the new Figure 1.

- I would consider adding a scheme with how blocks, windows and bSFS are related as a figure or a supplement. It would help the reader to understand the underlying logic of the approach.

An excellent suggestion! We have added a new figure (Fig 1) to better explain how blocks are sampled and how they relate to windows.

I understand the logic of doing a 2-step estimation. However, I wonder whether the authors could test **the reliability of this approach** through subsampling or jackknifing of the different chromosomes. How variable is the estimate of T, since further down analysis relies on it being a global parameter.

This is a good point and we have expanded on this to better explain our rationale (see also responses to comments from reviewer 2 below). The idea to subsample different chromosomes is interesting, but comes with its own problems: given the observation that the number of strong barrier regions differs widely between chromosomes and given that parameter estimates for IM models are partially confounded, we would expect some variation in *T* estimates based on different chromosomes. More importantly, our analysis of the Heliconius dataset shows that the information about *T* is rather limited overall in this dataset and that the global estimate of T has very little effect on the *m_e* scan. In other words, while we believe conceptually (and in general) that the two step inference procedure is the correct approach, for our test dataset the first step does not matter much: analysis under a migration-only model (*T* -> infinity) gives a very similar *m_e* landscape. We have made this clearer in the text (l. 450 following).

It is unclear to me how sensitive the method is to the mutation rate. I am wondering how an increase/reduction in local mu may be reflected on the local estimates of m or N.

This is an interesting point. We argue that setting up a comprehensive sensitivity analysis that explores the effect of mu heterogeneity is beyond the scope of the paper. While we know very little about how mu varies along genome, mutation accumulation experiments (e.g. Ness et al 2015, GenomeResearch) have so far found no evidence for rate heterogeneity over the scale that would be relevant for our analysis (gIMble windows span 100kb). In other words, we would argue that heterogeneity in mu between windows is biologically implausible and therefore irrelevant. On the other hand, there is a lot of data to suggest that mu varies over the scale of gIMble blocks. Moreover other aspects of real genome data (mis-annotation, mapping errors etc.) are likely to affect the window-wise tallies of bSFS configurations in similar ways to fine-scale mu heterogeneity. Such heterogeneity in mu would lead to global biases in parameter estimates which we now discuss (l. 525).

I wonder how much the grid has an impact on the result. I understand the computational burden and the impossibility of using a larger grid, but I wonder how robust the estimations obtained are to a subsampling of the grid (since the grid is precomputed, it should be doable).

We have resisted the temptation to base the analysis on the largest possible grid, i.e. the analysis we present is actually based on a relatively small grid (in terms of computational effort). A finer grid would be very possible but will simply yield more finely resolved local parameter estimates. In our experience the likelihood surface under the IM model is smooth and has a single global optimum. Given this, we do not believe that finer resolution of local parameters would be very interesting. In other words we have no reason to expect a finer grid to impact any of our results. We have added more detail to explain the choice of the grid (l. 272).

Finally, I wonder whether the authors could discuss how dependent the approach is to having gene

flow in one direction only. In the example provided, backcrosses in one direction are impossible, but what happens if one direction is negligible compared to the other. Lets call m12 migration in the "main" direction and m21 migration in the opposite direction. Here m21=0. But what happens if m21= 0.01 m12. Is the method still applicable? What about m21=1/1000 m12 or 1/10?

This is a good point; we have now added a paragraph to discuss the impact of bi-directional migration (see also our responses to reviewer 3) (l. 509-517)

Minor comments:
L178: "Crucially, we assume that blocks are only indirectly affected by selection at nearby linked sites and otherwise evolve neutrally and under a constant mutation rate. While these assumptions allow us to treat blocks within the same window as statistically exchangeable, they necessitate a careful and consistent filtering strategy for variant and invariant sites." I am a bit confused here. LD is still going to decay as a function of the distance from the selected sites, especially if genes can only be found outside windows (and not within but skipped).

Apologies for the misunderstanding about how windows are defined (genes can be within windows but are masked/excluded from the analysis). Given the new methods section this is hopefully now clear.

L208 and 209: are windows defined over 50,000 or 500 blocks?

Windows are defined over 50,000 pair-blocks. We have explained better how this gives a minimum window span of 500 times the block length.

L208: I would precise here that by offset, the authors mean that the window is moved by 10,000 pair blocks (and therefore windows overlap) and not by 60,000 pair blocks (and windows don't overlap).

Thanks for this suggestion. We have added text to make this unambiguous (l. 240).

L242-243: Could this variation not be explained by changes in recombination or mutation rate?

Heterogeneity in recombination rate IS included in the simulation so this is not a possible explanation. We have made this clearer in the text. mu would have to vary by more than a factor of two to explain Ne variation (see our responses to the comment above).

L247: is this not always true, even if there were no local variation in any of the parameters of the model. The local fitting simply captures part of the noise in that case. If you were to do the fitting of the parametric bootstrap data (if doable), using the same approach allowing for local variation, how much would the fit of the model be improved?

This is a good point. We have tried to make it clearer that we have indeed fitted the parametric bootstrap data to a model of varying *N_e* and *m_e* in the same way as the real data.

L 255: it is unclear to me how conservative this "normalization" is. Assuming Ne was truly constant, how much of the variation due to changes in local me would be captured by this approach?

While our approach may be overly conservative, i.e. by allowing for *N_e* variation we soak up some of the true variation in *m_e* we do not see this as a problem. We would argue that our assumption that *N_e* is variable both under a model of background *m_e* and reduced *m_e* is much more biologically realistic than the opposite assumption (of a truly constant *N_e*). In other words, the question of how DeltaB would behave under a scenario where there was no variation in *N_e* but variation in *m_e* seems irrelevant for analysing real data given the very strong support for *N_e* variation in our data (and in genomic datasets in general).

Figure 4 : what is an effective length?

This was confusing and we have changed the wording here (from “effective length” to “minimum window span”). We also added a reference to FigS1 to make this clearer.

Figure S1: what are na and nb?

na and nb are the sample size, i.e. the number of individuals sampled from taxon a and b respectively. We have changed the caption to make this clear.

Reviewer #2

Speciation genomic studies primarily aim to understand the genomic architecture of barriers to gene flow, including the number, distribution and effect size of barrier loci. Achieving these aims requires that researchers are able to quantify how rates of gene flow vary across genomes, while also accounting for the diverse array of other factors that can shape patterns of variation. Methodologies for addressing these aims have lagged far behind access to data. In this manuscript, Laetsch et al. outline and implement a new model-based inference scheme that represents a significant leap beyond what is currently available.

This is a really beautiful paper that not only introduces the method and how to use it, but applies it to a well known study system. I can really see that the authors have tried to make this paper accessible to broad readership, but in revising it, I would urge them to take a step back and really think carefully how they would explain things to a new researcher entering the field. You think of the following comments as a set of clarifications, which the authors could try and expand on.

Many thanks for these clear and extremely constructive comments

Minor comments:

Line 94: maybe replace with ‘so does not reveal the locations of barrier loci in the genome’, which is a bit clearer than ‘does not allow scanning for barriers to gene flow’.

Changed as suggested.

Paragraph 69: I think it would be worth pointing out that efforts have been made to gauge heterogeneity in m_e (i.e., by modelling multiple classes of loci with different rates of m) while trying to control for confounding factors (i.e., by modelling classes of loci with different N_e), but this is crude and again does not reveal the locations of barrier loci.

A good point. We have added text (and additional references) to explain this better. We had previously only explained this in the section “Demographically explicit genome scans” but now mention this work in “model-based demographic inference”.

Line 96: ‘an awkward choice’, makes it sound like researchers do one or the other, when many researchers just do both things (i.e., most researchers see them as complementary rather than as alternatives), and then interpret the results in context of one another (i.e., comment on both the population processes and the heterogeneity). So this becomes a bit of a weak justification for why we would ideally like to reconstruct a fully specified model of the speciation process.

The point we wish to make here is not to justify estimating a fully specified model of the speciation, but to highlight the fact that truly joint analyses that include heterogeneous demography and scans for

barriers along the genome are so far not possible. We have changed the text to explain this better (l. 101-107).

Line 109 on: this is the detail that I thought was missing from the section ‘model-based demographic inference’ above.

We agree that it makes sense to highlight the substantial previous efforts to model heterogeneity in m_e (Roux et al, 2013 & Sousa et al 2013) earlier.

Line 114: delete ‘alternative’.

Done

Line 156: invites the question ‘is variation in m_e correlated with recombination rate variation.

Good point, the assumption that m_e is correlated with recombination is implicit; we have modified the text to make this clearer.

Line 169: conditioning the inference on global estimate of ***T*** is a reasonable simplification in a complex analysis like this. However, given that speciation often proceeds through selection on old variation, it makes me wonder when this would break down and what parameters might be affected. For example, old inversion polymorphisms or introgressed alleles may have much deeper divergence than the rest of the genome in a local speciation event. I guess situations like this would be unlikely to affect the detection of barriers but would likely impact the estimates of m_e?

This is a good point which made us realise that we should have made it clearer that the analysis for barriers of the *Heliconius* dataset is robust to the assumption of a global *T* : i.e. for this dataset an inference under a migration-only model gives a nearly-identical *m_e* trajectory. In other words, while we agree with the reviewer's assessment that, conceptually, a two step analysis which conditions on a global *T* is a reasonable/logical approach, it turns out that step 1 does not matter for this particular dataset. We have now made this clearer (l. 406). We also highlight the fact that gIMble can be used to infer parameters and models for any data partition which allows researchers to test whether *T* differs between known inversions and the genome-wide background. There are no inversion polymorphisms or fixed differences known for the focal *Heliconius* taxa, but see Poikela et al 2023 https://www.biorxiv.org/content/10.1101/2022.11.15.516589v3 for such an analysis in *Drosophila*.

Fig 1: this is really helpful. But I wonder if you could add a simple cartoon of a demographic model here so less seasoned readers can see how the parameters map onto it.

Following this comment and those from the other two reviewers we have replaced this figure with a new figure which explains what parameters are inferred along the genome rather than the bioinformatic detail of the workflow. Our new figure 1 includes a cartoon of the demographic model and parameters. The previous figure 1 is relegated to the Supplementary Material.

Line 199: ‘we focused all analyses on intergenic sequence defined as any sequence without gene or repeat annotation’. Can you explain why you don’t you don’t include variable sites with gene annotation?

We have added more explanation (l. 183-189) to justify our choice of data partition.

Line 200: ‘For the sake of simplicity, we ignore the Z chromosome, which is known to have greatly reduced genetic diversity and gene flow in Heliconius taxa’. Can you explain why this is simpler? Seems like an interesting region to include because there are some predictions how N_e would vary compared with other LGs.

We have added text to better explain this choice (l.219-224).

Line 202: are the saying that the pair blocks all contain 64 variant sites when you say ‘bases’? Worth trying to keep this consistent. If so, what is the mean length, s.d., min, max of the pair blocks?

This is a really important point which we clearly failed to explain well enough. We have expanded on this in the new subsection “Summarising genomic variation in blocks and windows” (l. 180) to explain that pair-blocks i) are defined for a strict length of bases (which includes both invariant sites and variants) and ii) may include gaps (see also our new Figure 1).

Line 219: So gene flow in the global model, migration only occurs in 1 direction (i.e., kind of a source sink model). Is this an intrinsic limitation of the method, or is it just something you decided to do here to keep the number of estimated parameters low? Also, I wonder if it’s useful to show fig S4 in the main text, perhaps coupled with table 2.

Unfortunately unidirectional migration is a hard limitation of the current method. We have added a paragraph (l. 510-517) discussing the likely effect of model misspecification (see responses to reviewer 1). We decided to keep Fig S4 as a supplement, given that it contains the same info as the table but takes up a lot of space. Our new Fig 1 panel (i) now includes a cartoon of the IM model we are fitting.

Line 262: what do negative values of $\Delta B,0$ mean?

We have expanded on this (l.304) to make it clear that negative values of $\Delta B,0$ simply mean greater local support for a model with the background level of gene flow compared to a no-gene-flow scenario, i.e. a complete barrier.

Fig 3 results. I’m surprised that so much of the genome shows really negative $\Delta B,0$. Based on the explanation, I would have expected most windows to be hovering around ‘sea level’, and barrier regions to rise above it (which they do). Clarifying the above point will help.

Given the explanation above, the fact that most of the genome shows negative values of $\Delta B,0$ is unsurprising.

Fig 5 caption: “the prediction under a polygenic model (modified from [9]) is shown in green”. This needs more context.

Yes, this was very cryptic. We have added more explanation in the caption to make this clear.

Fig. 7. Not a very intuitive layout (i.e., comparing x-axes between plots arrayed horizontally.). Each row would be better presented as a separate boxplot box/dot plots, so that the distributions could be more easily compared.

Many thanks for this suggestion. We agree that a set of violin-plots makes more sense and have replaced this figure as suggested.

Line 356: this is a really fun section. Going into it, I really thought the answer would be counting up the number of barriers identified by Gimble. I think you need to start this section, explaining why it does not make sense to do this, because I think users of the program may be tempted to.

This is an excellent suggestion! It took us some thinking to resist this temptation. We have rewritten the start of this paragraph to make this clearer (l. 401).

412-415: this is more clearly stated than in the introduction.

We have tried to transfer some of this clarity into the newly worded intro.

449: As I mentioned, I think this assumption should be stated way earlier.

Agreed, we state this assumption in l. 165 and Fig1.

491: Again this kind of explanation was needed near line 356.

We have added this explanation (l. 226) to make this assumption clear from the outset.

Reviewer #3

The gIMble approach has already gained considerable popularity in the population genomics community over the last few years, and its publication is eagerly awaited (as it has already been used/approved by some). So I'm not going to delay its publication, or create a false suspense in my review: the article must be published, I support it 100%.

methodological dissemination
Having said that, I have my doubts about the current form of the article, particularly in terms of making it more accessible to non-methodologists. In my opinion, the article lacks a textbook-style figure. For example, DaDi is so popular because of the visual simplicity of the original article that, in 2023, everyone associates SFS with DaDi, simply because the link between demographic history, observed SFS and computational inference is accessible to the greatest number of people (even if the heart of the method is a black box for many readers).Of course, Figure 1 is an attempt at a synthesis, but I don't understand it: what's a grid? What's the difference between green, orange, blue and purple? Why are there dotted lines between the purple grids? What does the gIMble store actually contain? What are orange, blue and violet spectra? Etc… This figure is too 'bioinformatician' and didn't interest me; I think it would be better placed in a Readme. On the other hand, given that the authors are using a lexical corpus that is not very widespread in the literature, I think it is important to explain better what a tally is, what a grid is, the link between tally and bSFS, and how a barrier bSFS is different from a non-barrier bSFS. Of course, this is mentioned in the text, but instead of the current figure 1, I can imagine a figure that would be used in presentations by people using gIMble, a more schematic figure based on an alignment of sequences obtained in species A and B. Part of this alignment is linked to a barrier (central part, for example), and the rest is not linked to a barrier. Then to show the reader graphically and sequentially how gIMble handles such an alignment. This will give the reader a better understanding of what a block, a grid, a tally and a bSFS are, and how these elements can be used to distinguish a barrier region from a non-barrier region. This is just a suggested figure, but I think it will increase the impact of the article more than the current figure 1, which doesn't really help you understand how gIMble works (it's more about the workflow architecture with a black box at its centre).

We thank the reviewer for these comments! Having lived on the inside of gIMble for the last few years, it has made it difficult for us to see how to best explain the key concepts to a new user and we agree completely that this is exactly what Figure 1 should achieve. Given that the bSFS is a higher dimensional data summary than the 2D SFS, the link between data and evolutionary process is of course harder to explain than for dadi, but our new Fig 1 is an attempt to visualise how the bSFS captures information about the model.

Practical application to empirical data
The application with the _Heliconius_ data is well written overall, but some additional information could be added to help the future user make choices.
1. In particular, how do you choose the length of the blocks in nucleotides? Why/how did the authors

choose 64? Is this an important parameter in the analysis, and how would the results differ with different block sizes?

This is an important point and we agree that our choice of block length needs to be explained. We have now added parameter estimates for longer and shorter blocks (Table S3) to highlight that the inference is robust to the choice of block length (l. 264). We have also added text (l. 228) to explain our choice of block length which involves a trade-off between power (which increases with l) and bias (which also increases with l). Our choice of l = 64 corresponds to an average of one heterozygous site (in each of the two taxa) per block which is a useful compromise for organisms with $\rho \sim \mu$.

2. how flexible is gIMble with regard to the scenarios to be compared, i.e. which scenarios will users actually be able to compare? For example, is bidirectional introgression really impossible to study (with asymmetric barriers)?

Yes, it is currently not possible to study bidirectional introgression. We have made this clearer and have added a paragraph discussing the effect of bi-directional migration (l. 501-517).

3. fig. 2, grey panels: why doesn't it look like I have 16 bins between 0 and 1e-6?

Many thanks for catching this! We have updated the figure (to include high m bins that were not plotted in the previous version).

4. concerning the choice of thresholds for the migration rate, 0.2 times the average rate corresponds roughly to 0.08 migrants per generation in the _Heliconius_ example.

We apologise for the misunderstanding. We are varying m_e, not M. Given that M is around 1.5 migrants per generation (see Table 1) our threshold of $m\_e^* = 0.2$ m_e corresponds to 0.3 migrants per generation on average.

But in some cases, 0.2 times m_e may correspond to a greater number of migrants per generation, whose effects on coalescence are the same as 1 times m_e. In order to define the m_e threshold, shouldn't readers be given a threshold expressed directly in terms of the number of migrants per generation?

We agree that it would make sense in principle to define m_e thresholds in terms of migrants per generation (4 N_e * m_e). However, in practice, such a scaling makes little sense given that we choose to fit a model where N_e parameters (in both the daughter pops and the ancestral population) are allowed to vary. Thus, we believe that in practice the more sensible choice is to define barriers in terms of the relative (to the background) reduction in m_e (rather than M). This seems to be an important difference to how heterogeneity is modelled in DILS and we have added text to make this clearer (l. 177).

5. lines 275-280: for me, the fact that barriers are not just Fst outliers is a very important message to get across to the community. I'm dreaming of a synthesis article where this would be highlighted for different types of living organisms (plants, animals, fungi).

We couldn’t agree more and have highlighted this throughout the MS.

6. In the "Robustness to model choice" paragraph, without wanting to come across as a tiresome reviewer with all the potential biases, it is difficult to ignore the secondary contacts. I think that future users need to be reassured here about the impact that such a model would have on gIMble inferences. This can also be done by providing information on how to process such a dataset with gIMble.

We have expanded the Discussion (l.518) to provide concrete guidance. Unlike other model violations/misspecifications (see our response to mu heterogeneity and bidirectional gene flow) we

agree that it is unclear how meaningful gIMble scans on data generated under a secondary contact model would be. While for our test data there is a strong prior for the IM model over a model of secondary contact (see Martin et al 2013, Rosser et al 2015), we caution users to assess the support for the IM model over alternative scenarios.. Based on the comparisons we performed between an IM model and a model with infinite split time, we found that the difference between these models did not matter for the purpose of detecting barriers. That is, there is a wide range of parameter space in which barriers are detectable with gIMble. However, this may look very different under a model of secondary contact or a model of isolation with initial gene flow (IIM). In this case we expect that the combination of T and the length of the period of gene flow to have a strong impact on the power to detect barriers. We discuss these issues in the new version (l. 522).

7. without checking whether this is the case or not, if it isn't, I think it would be useful if the VCFs (or fasta) used to analyse the _Heliconius_ data could be downloaded directly rather than having to redo all the bioinformatics. This dataset is perfect for users to get to grips with the tool, or to compare gIMble results with other t.q DILS methods, for example.

Following this suggestion we have uploaded the assembly FASTA and the vcf files to data dryad. While this makes it easier to replicate our analysis, we stress that it might be more useful for most readers/users to test-run gIMble on a small test dataset which we provide as part of our release.

8. Finally, a more personal question: I'm currently interested in comparing speciation dynamics between contrasting groups of organisms (e.g. plants _versus_ animals; internal _versus_ external fertilization; parasites _versus_ non-parasites).
How optimistic would the authors of gIMble be about applying this tool to such analyses involving several dozen pairs of species?

This comparative analysis sounds great and we are very happy to talk to the reviewer about this.

Once again, I have only superficial comments to make, as I'm already convinced of the relevance and quality of the tool. I simply think that this article needs to be made more like a textbook than a semi-readme, which is currently too technical to understand the basics of gIMble, and also too superficial to really do a step-by-step analysis (which is not the aim of a scientific article).

Many thanks for these very constructive comments.
